# Clinical translation of [^18^F]ICMT-11 for measuring chemotherapy-induced caspase 3/7 activation in breast and lung cancer

**DOI:** 10.1007/s00259-018-4098-9

**Published:** 2018-09-27

**Authors:** S. R. Dubash, S. Merchant, K. Heinzmann, F. Mauri, I. Lavdas, M. Inglese, K. Kozlowski, N. Rama, N. Masrour, J. F. Steel, A. Thornton, A. K. Lim, C. Lewanski, S. Cleator, R. C. Coombes, Laura Kenny, Eric O. Aboagye

**Affiliations:** 1 Department of Surgery and Cancer, Imperial College London, Hammersmith Hospital, Du Cane Rd, London, W120NN UK; 2 Department of Radiology, Imperial College Healthcare NHS Trust, London, UK; 3 Department of Computer, Control and Management Engineering Antonio Ruberti, University of Rome, La Sapienza, Italy; 4 Department of Oncology, Imperial College Healthcare NHS Trust, London, UK

**Keywords:** Apoptosis, Positron emission tomography, [^18^F]ICMT-11, Isatin sulfonamide, Caspase-3

## Abstract

**Background:**

Effective anticancer therapy is thought to involve induction of tumour cell death through apoptosis and/or necrosis. [^18^F]ICMT-11, an isatin sulfonamide caspase-3/7-specific radiotracer, has been developed for PET imaging and shown to have favourable dosimetry, safety, and biodistribution. We report the translation of [^18^F]ICMT-11 PET to measure chemotherapy-induced caspase-3/7 activation in breast and lung cancer patients receiving first-line therapy.

**Results:**

Breast tumour SUV_max_ of [^18^F]ICMT-11 was low at baseline and unchanged following therapy. Measurement of M30/M60 cytokeratin-18 cleavage products showed that therapy was predominantly not apoptosis in nature. While increases in caspase-3 staining on breast histology were seen, post-treatment caspase-3 positivity values were only approximately 1%; this low level of caspase-3 could have limited sensitive detection by [^18^F]ICMT-11-PET. Fourteen out of 15 breast cancer patients responded to first–line chemotherapy (complete or partial response); one patient had stable disease. Four patients showed increases in regions of high tumour [^18^F]ICMT-11 intensity on voxel-wise analysis of tumour data (classed as PADS); response was not exclusive to patients with this phenotype. In patients with lung cancer, multi-parametric [^18^F]ICMT-11 PET and MRI (diffusion-weighted- and dynamic contrast enhanced-MRI) showed that PET changes were concordant with cell death in the absence of significant perfusion changes.

**Conclusion:**

This study highlights the potential use of [^18^F]ICMT-11 PET as a promising candidate for non-invasive imaging of caspase3/7 activation, and the difficulties encountered in assessing early-treatment responses. We summarize that tumour response could occur in the absence of predominant chemotherapy-induced caspase-3/7 activation measured non-invasively across entire tumour lesions in patients with breast and lung cancer.

**Electronic supplementary material:**

The online version of this article (10.1007/s00259-018-4098-9) contains supplementary material, which is available to authorized users.

## Background

Cell death, is recognised for the constant regulation and harmony of biological systems and is known to occur by several mechanisms, predominantly necrosis and apoptosis. Necrotic cell death is characterised by a non-specific process of events resulting in plasma membrane rupture and a localised inflammatory response around the surrounding cells and tissues. Apoptosis (programmed cell death, first described by Kerr and colleagues [1, 2]), however, is a precise series of well-executed events with upstream regulators and downstream effector components [3, 4], leading to the systematic dismemberment of the cell by “apoptotic triggers”. The “apoptotic triggers” are kept in balance by pro- and anti-apoptotic regulatory proteins, including members of the Bcl-2 family [3]. One of the key players in the execution of apoptosis is a family of caspases (cysteine aspartate specific proteases) [4]. Caspases 8 and 9, known as the initiator caspases, are responsible for initiating a cascade of proteolysis by cleavage of pro-caspases 3, 6, and 7 to their activated form. The controlled demolition of cellular components resulting in DNA fragmentation is unique to apoptosis, and one that is driven by caspase 3, a central effector caspase. It is this unique feature of caspase-3 that permits its potential use as a non-invasive biomarker of apoptosis [5].

Imaging the apoptotic process may prove to be invaluable for the following reasons: a) by enabling anti-cancer therapy response assessment at earlier time-points than current response criteria allows with conventional imaging, apoptosis imaging may aid the decision to implement changes to treatment in the context of drug resistance sparing the unwanted side-effects of ineffective treatment, and b) secondly, by allowing for the pharmacodynamic assessment of drugs that target the apoptotic machinery in early-phase trials during drug development.

There has been a handful of pre-clinical and clinical PET imaging studies attempting to image molecular and biochemical events of the apoptotic process [6–14]. Studies with [^18^F]ML-10 [9, 15–17] and [^99m^Tc]Annexin V [11, 12, 18–20] showed promising results in humans. A study of [^18^F]ML-10 [9] — a member of the aposense family of biomarkers that measures ‘apoptotic imprint’ — in human subjects reported favourable dosimetry and biodistribution, and binding to apoptotic sites in testicular tissue of mice, confirmed by terminal deoxynucleotidyl transferase (TdT) dUTP nick-end labeling (TUNEL) of apoptotic cells; initial studies reported correlation of early changes of tumour [^18^F]ML-10, and later changes in anatomical tumour dimension following radiotherapy [16, 17].

Annexin V, a 36 kDa calcium-dependent protein with the ability to bind to cells during all stages of the apoptotic process, has high affinity for phosphatidylserine (a phospholipid, normally located on the inner leaflet of cell membranes). During apoptosis, phosphatidylserine is exposed to the extracellular surface and provides an opportunity for annexin V binding. To date, [^99m^Tc]HYNIC-annexin V has been investigated widely for the imaging of apoptosis, and has provided invaluable information in several disease settings [11, 12, 18, 19]. Both [^18^F]ML-10 and [^99m^Tc]Annexin V imaging, however, pose limitations. [^18^F]ML-10 remains undefined in its specific target and [^99m^Tc]Annexin V in its non-specific uptake, which has proved difficult in distinguishing between apoptosis and necrosis.

Clinical studies evaluating apoptosis in breast cancer therapy have shown an increase in apoptosis within biopsies taken at 24 h post-chemotherapy (comparing six cycles of epirubicin, cisplatin, and fluorouracil with six cycles of doxorubicin and cyclophosphamide) compared to baseline [21–24], as well as at 48, 72, and 96 h post-treatment (combination of epirubicin, cisplatin, and fluorouracil, doxorubicin and cyclophosphamide and weekly paclitaxel) [25]. In the small numbers of patients studied, the authors highlighted the wide variation of changes in apoptosis and or/necrosis and importantly, no correlation of the changes with clinical response to treatment.

**Fig. 1:**
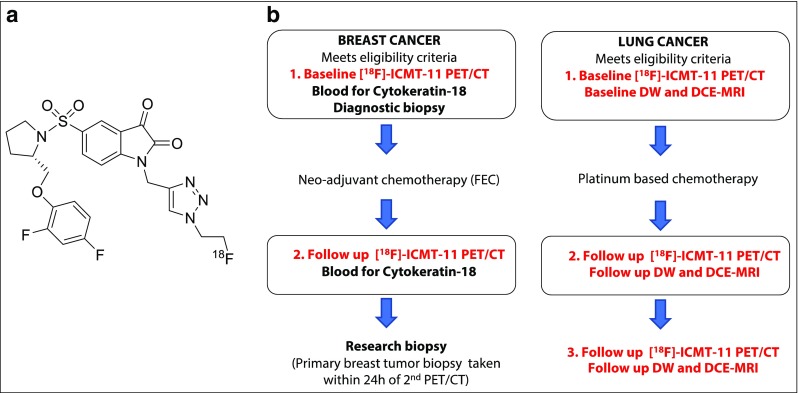
Chemical structure of [^18^F]ICMT-11 and) study design in breast and lung cancer cohorts. **a** Schematic diagram of chemical structure of [^18^F]ICMT-11. **b** Study design in breast cancer patients receiving neo-adjuvant chemotherapy. Patients underwent a baseline and follow-up scan with a repeat second breast biopsy under USS guidance, within 24 h of the second PET/CT scan. The cohort of lung cancer patients recruited to study all received first-line chemotherapy (combination chemotherapy with a platinum-based compound — Cisplatin). [^18^F]ICMT-11 PET/CT and MRI (DW and DCE) were performed at three time-points (baseline, follow-up at 24–48 h, and within 6–8 days post-chemotherapy. *FEC* = 5-fluorouracil, epirubicin and cyclophosphamide, *USS* = ultrasound, *MRI* magnetic resonance imaging, *DW* = diffusion-weighted, *DCE* = dynamic contrast-enhanced

Breast cancer is, to an extent, the ideal clinical setting to obtain tissue pre- and post-therapy. Biopsies offer a snapshot of caspase activation at microscopic levels; however, PET and MRI provide dynamic and perhaps more robust non-invasive methods for assessment of apoptosis across the entire lesion volume. The fundamental requirements of any PET radiolabeled probe are ease of synthesis, robust and reproducible facile radiolabelling procedure, and high specificity and selectivity. [^18^F](S)-1-((1-(2-fluoroethyl)-1H-[1,2,3]-triazol-4-yl)methyl)-5-(2(2,4-difluorophenoxymethyl)-pyrrolidine-1-sulfonyl), [^18^F]ICMT-11 (Fig. 1a), an activated caspase-3/7 specific PET imaging radiotracer, was designed from a library of isatin-5-sulfonamides, a chemical class known to have caspase inhibitory activity. With regard to the mechanism of action in relation to selectivity for binding activated caspase-3/7, the dicarbonyl functionality of isatin sulfonamides, including [^18^F]ICMT-11, is thought to form an intracellular enzyme–tracer complex with the cysteine residue of the active site of caspase-3/7 — forming a thiohemiketal via the electrophilic C-3 carbonyl of the isatin sulfonamide and the nucleophilic cysteine thiol functionality [26]. [^18^F]ICMT-11 was selected for further evaluation, due to its subnanomolar affinity for activated caspase-3, high metabolic stability, reduced lipophilicity, and facile radiolabelling [27]. Here, we report the results of the first clinical study investigating [^18^F]ICMT-11 as a non-invasive biomarker to assess tumour apoptosis in locally advanced breast cancer pre- and post-first-cycle of neo-adjuvant chemotherapy (NCT), and in locally advanced lung cancer patients receiving chemotherapy as first-line treatment.

## Materials and methods

### Radiopharmaceutical preparation

[^18^F]ICMT-11 synthesis and radiolabelling was performed by Imanova Ltd., as previously described [28].

### Patients

Two independent phase 1 non-randomised open-label prospective feasibility studies (breast and lung cancer) were recruited from 2013 to 2016. The aim of this study was to assess the effect of chemotherapy on [^18^F]ICMT-11 uptake by PET and relationship between this variable and blood and tissue activated caspase-3/7 markers. Inclusion criteria in the breast cohort required patients 18 years or older, newly diagnosed with locally advanced potentially operable breast cancer receiving NCT followed by surgery. At least one measurable breast lesion ≥ 20 mm was required on conventional imaging. Patients were excluded from the study if they had received any chemotherapy, immunotherapy, biologic therapy, or investigational therapy within 14 days prior to the first dose of [^18^F]ICMT-11 injection. Exclusion also applied if the subject was undergoing occupational monitoring of ionising radiation exposure, was lactating or pregnant, or was taking any anticoagulation therapy, a prolonged prothrombin time, or had a positive Allen’s test.

All breast patients, as per standard routine diagnostic and staging procedures, had a pre-treatment core-needle biopsy and ultrasound (USS) for histological confirmation with characterisation of hormone receptor status alongside imaging with mammogram, and if indicated an MRI of the breasts. Patients had a sentinel lymph node biopsy prior to NCT if deemed appropriate by their clinical team. NCT consisted of six cycles of FEC-T (5-fluorouracil, epirubicin, cyclophosphamide and docetaxel) alongside trastuzumab (Herceptin) in those with HER2-positive breast cancer. Patients were treated at Imperial College Healthcare NHS Trust, and one patient was treated at their local oncology unit. Clinical response using RECIST 1.1 after three cycles of NCT using USS and after six cycles of chemotherapy on surgical histopathology specimens (post wide local excision or mastectomy ± axillary lymph node clearance) was documented. Patients underwent a baseline dynamic [^18^F]ICMT-11 PET/CT prior to start of NCT for 65 min followed by a repeat PET/CT scan performed 24–48 h (early) or within 2–14 days (late) post-chemotherapy and repeat breast biopsy within 24 h of the second PET/CT to correlate apoptosis in breast tissue utilising cleaved (active) caspase-3 staining by immunohistochemistry.

In the small cohort of lung patients (*n* = 2), subjects diagnosed with non-small cell lung cancer undergoing platinum-based chemotherapy treatment (both patients were treated with pemetrexed and cisplatin) were enrolled. At least one measurable lung lesion ≥ 20 mm was required on conventional imaging. Patients were required to have a PET/CT, as well as DW- and DCE-MRI at baseline and post-chemotherapy (24–48 h (early) and within 6–8 days (late) post-treatment). A baseline DW- or DCE-MRI over the area of interest was followed by a 61-min dynamic [^18^F]ICMT-11 PET/CT scan.

The Harrow and Westminister London Research Ethics Committees approved the breast and lung study, respectively. All subjects signed a written informed consent form. The study was conducted according to the Declaration of Helsinki. The administration of radioactivity was approved by the Administration of Radioactive Substances Advisory Committee, U.K.

### Imaging acquisition

Images were acquired on a Biograph 6 TruePoint PET/CT scanner (with TrueV; extended field of view [Siemens]) with 21.6 cm axial and 60.5 cm transaxial fields of view. Patients in both studies underwent an attenuation CT scan (CT settings: tube potential, 130 kV; exposure, 15 effective mAs; pitch 1.5; slice thickness, 5 mm; rotation time, 0.6 s; effective dose of 2.5 mSv) of the thorax before administration of [^18^F]ICMT-11 injection. [^18^F]ICMT-11 was injected with a target dose of 300 MBq (maximum dose 370 MBq) as a slow bolus in 1–20 mls of saline over 30s. Dynamic PET imaging was performed in a single bed position over 65 min with blood and plasma radioactivity measurements at specified time-points. Data were binned into 35 frames and reconstructed using the ordered subset expectation maximization algorithm (3 iterations and 21 subsets). In the lung cohort, dynamic PET imaging was performed in a single bed position over 61 min. Data were binned into 36 frames and reconstructed using the ordered subset expectation maximization algorithm (3 iterations and 21 subsets). Radioactive blood data were taken but were not analysed in this study.

### Image analysis

All volumes of interest on PET/CT were outlined manually on Hermes (Hermes Diagnostics, Stockholm, Sweden) by a single investigator (SD) to avoid any interobserver variation. For the breast study, volumes of interest (VOIs) were drawn on fused PET/CT datasets by outlining the whole primary breast tumour and any involved axillary lymph nodes [regions of interest (ROIs) over several slices]. VOIs were also drawn using a 2-cm fixed sphere to outline contralateral breast tissue, normal lung, bone, muscle, and aorta. In the lung study, VOIs were drawn outlining the primary lung tumour lesion, normal contralateral lung, bone, muscle, and aorta. SUV_60ave_, SUV_60max_, tumour to breast ratio (TBR_60max_) and tumour to muscle ratio (TMR_60max_) were obtained at baseline and post-chemotherapy in both studies.

In both studies, all VOIs were also outlined on fused PET/CT images to create binary object masks using Analyze software (version 11; Biomedical Imaging Resource, Mayo Clinic). The object masks created for tumour and lymph nodes, alongside respective dynamic PET data, were used within Matlab 16a (The MathWorks®) for analysis and characterisation of the VOIs by voxel intensities sorting. Voxel-wise analysis of PET data was done as previously described [29]. Briefly, this involved extraction of all the voxels within each VOI and sorting as per their intensity frequency. PET-based voxel intensity sorting (PVIS) identifies any shifts in voxels, as would otherwise be difficult to observe by using only ROI analyses, where any spatially discrete areas of effect may be averaged. Any shifts observed (higher intensity voxels) were presumed to be in keeping with apoptosis and shifts to lower intensity voxels representing necrosis. The highest voxel intensities (taken as a cut-off at the 95th percentile) are more indicative of high radiotracer retention than the voxel mean, and were taken to biologically represent apoptotic cells.

Histogram analysis was then performed using in-house software developed in Matlab 15a (The MathWorks^(R)^), to calculate first-order statistics.

### DW-MRI and DCE MRI

DW-MRI and DCE-MRI image analyses is described in Supplementary materials and methods 1).

### Cytokeratin-18 measurements

Blood samples were taken in breast patients to measure CK-18. Two samples, each 7.5 mls, of blood from all patients were taken at baseline and at follow-up scan to measure M65 (measuring caspase-cleaved and intact CK-18) and M30 (measuring caspase-cleaved CK-18) using ELISA kits obtained by PEVIVA (BIOAXXESS, UK) (Supplementary material and methods 2).

### Cleaved (active) caspase-3 immunohistochemistry

A core biopsy was taken post-chemotherapy by a consultant interventional radiologist within 24 h of the second [^18^F]ICMT-11 PET/CT scan. All diagnostic and research breast biopsies obtained were processed as previously described [8] (Supplementary materials and methods 3).

#### Data and materials availability

All tissue stained for caspase-3 expression and cytokeratin analyses were stored with ECMC, Imperial College, London, UK. All materials received local tissue bank approval.

## Results

### Radiopharmaceutical

The radiolabelling of [^18^F]ICMT-11 was performed as previously described [28]. Radiochemical purity was > 99% on completion of synthesis with a mean (± SD) specific activity of 1373 ±1605 GBq/μmol (range, 199-9317 GBq/μmol) and pH of 5.02 ± 0.22 (range 4.57–5.66). The mean (range) doses injected in all breast patients pre- and post-chemotherapy were 335.6 MBq (278.4–353.3 MBq) and 343.1 MBq (282.4–359.1 MBq).

### Patients

The study was designed: (a) in breast cancer to assess the effect of chemotherapy on [^18^F]ICMT-11 uptake correlated with blood cytokeratin-18 assessment and biopsy-derived caspase-3/7 tissue expression within 24 h of the first dose of neoadjuvant FEC-T treatment, and (b) in lung cancer to measure the longitudinal effect of first-line chemotherapy on [^18^F]ICMT-11 correlated with diffusion-weighted and dynamic contrast-enhanced magnetic resonance imaging (DW- and DCE-MRI) at each time point; notably, the latter was more difficult to recruit to, as a result only descriptive data are presented. A total of 23 patients were recruited, 20 breast cancer patients and three lung cancer patients. Seventeen patients were evaluable (15 breast cancer patients, all of whom were female, and two lung cancer patients, both male). Five breast patients withdrew from the study (two due to tracer failure, two due to patient’s personal decision, and one due to needle phobia), and one lung patient also withdrew (due to extreme fatigue). Study design and patient characteristics are shown in Fig. 1b and Table 1 respectively. All breast patients had a diagnosis of invasive ductal carcinoma. Ten patients were found to have positive axillary lymph nodes on ultrasound (USS) and/or sentinel lymph node biopsy. All 15 patients completed a total of six cycles of FEC-T (three cycles of 5-fluorouracil, epirubicin, and cyclophosphamide, followed by three cycles of docetaxel). Four patients then commenced trastuzumab (Herceptin) concomitantly with docetaxel, after the first three cycles of FEC chemotherapy, for 1 year, as per standard local hospital guidelines. Fourteen patients received radiotherapy post-surgery, and one patient did not require radiotherapy. Oestrogen (ER), progesterone (PR), and human epidermal growth factor (HER2/neu) receptor status are documented in Table 1. Response was measured after three cycles of NCT by USS and after six cycles on histopathology at surgery. Fourteen out of 15 patients were classified as responders — partial response (PR) or complete response (CR) post-treatment — and one patient had stable disease (SD); no patient showed evidence of disease progression.

**Table 1:**
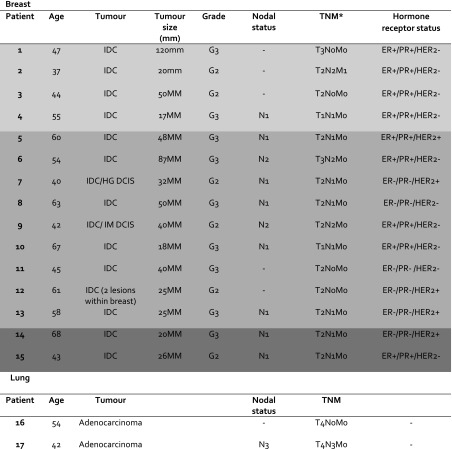
Patient characteristics

The median age (mean; range) of all patients: 54y (52; 37-67y), median weight 71.9 kg (73; 52.3-95.3 kg)

Light grey shading (patients 1-4, found to show a predominant apoptotic signature)

Mid-grey shading (patients 5-13, found to show a predominant necrotic and/or necrotic/apoptotic signature)

Dark grey shading (patients 14 and 15, found to show neither a predominant apoptotic or necrotic shift)

IDC Invasive ductal carcinoma, HG DCIS High grade ductal carcinoma in-situ, IM DCIS Intermediate grade ductal carcinoma in-situ, ER Oestrogen receptor, PR Progesterone receptor, HER2 Human epidermal growth factor receptor 2

*AJCC staging (7th edition) used for TNM staging in breast and lung cancer

Both lung cancer patients had a diagnosis of non-small cell carcinoma (adenocarcinoma). Clinical response in the patients with lung cancer were reported with computerized tomography (CT) using RECIST 1.1 [30] midway through their chemotherapy and at the end. One lung patient had a PR post four cycles of chemotherapy and proceeded to radiotherapy treatment (55Gy in 20 fractions over 4 weeks), the second patient, had a PR after three cycles and completed five cycles with SD after initial PR. This patient subsequently died due to infection, unrelated to tumour or treatment received.

### [^18^F]ICMT-11 uptake in breast tumours at baseline and post-chemotherapy

[^18^F]ICMT-11 was well tolerated by all patients, with no immediate or delayed complications observed. The median time (range) between baseline [^18^F]ICMT-11 PET/CT and start of NCT in breast patients was 6 days (1–14 days), and that between first cycle of NCT and post-treatment [^18^F]ICMT-11 PET/CT was 12 days (24 h–14 days).

**Fig. 2:**
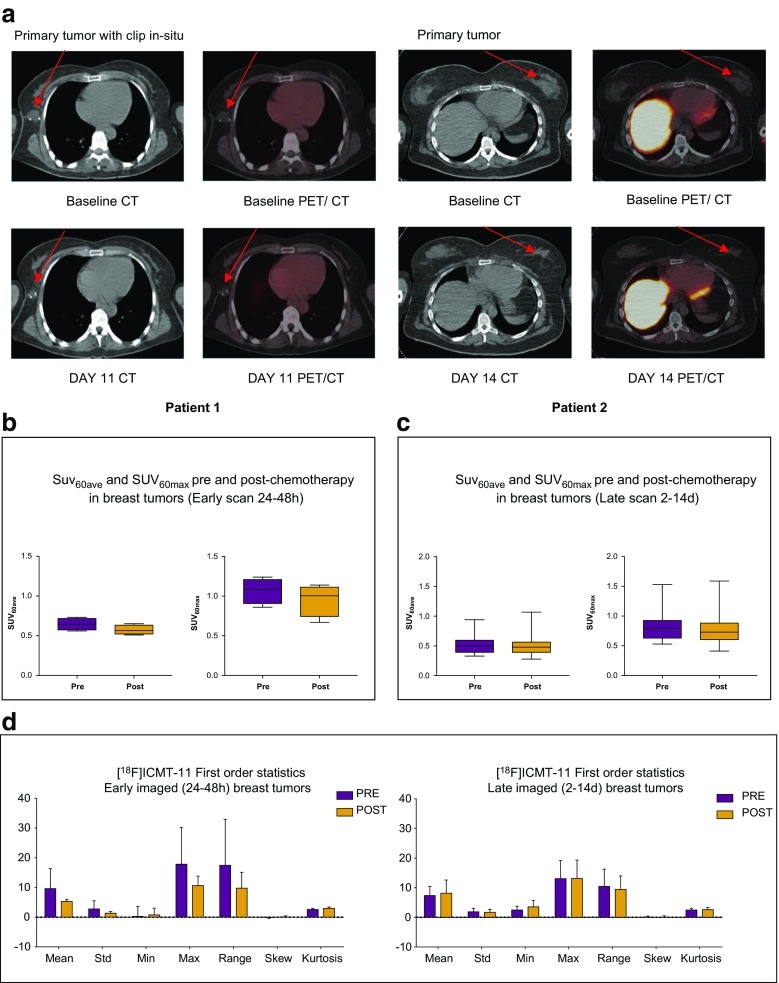
[^18^F]ICMT-11 uptake in primary breast tumours. **a** Axial CT and fused [^18^F]ICMT-11 PET/CT images of primary breast tumours in two patients, 1 and 2, at baseline (pre-) and post-chemotherapy. Low-level uptake is noted. **b** Pre- and post-chemotherapy SUV_60ave_ and SUV_60max_ values of breast tumours imaged at an early time-point (24–48 h) or **c** late time-point (2–14 days). Small changes were seen pre- and post-chemotherapy. **d** First-order statistics were extracted using in-house software under Matlab 15a [The MathWorks^(R)^], and a subset of features were selected to detect changes in early and late imaged breast tumours

All primary tumours and involved axillary lymph nodes were visible on conventional imaging and PET; however, not all lesions demonstrated uptake on [^18^F]ICMT-11 PET (Fig. 2a). For analysis, patients were divided into early (24–48 h) and late (2–14 days) imaging post-chemotherapy. There were four patients in the early imaging group and 11 in the late imaging group. The mean and maximum SUV at 60 min (SUV_60ave_ and SUV_60max_) pre- and post-chemotherapy are illustrated in Fig. 2b and c. The median (range) pre-treatment SUV_60ave_ and pre-treatment SUV_60max_ were 0.56 (0.33–0.94) and 0.91(0.53–1.24) respectively. Post-treatment SUV_60ave_ and post-treatment SUV_60max_ were 0.50 (0.38–1.07) and 0.75 (0.41–1.59) respectively. Pre- and post-chemotherapy ratios (± standard deviation, SD) of tumour-to-normal breast tissue (TBR_60max_), were 3.65 (± 1.71), 3.34 (± 1.52); corresponding tumour-to-muscle ratios (TMR_60max_) were 1.63 (± 0.43), 1.57 (± 0.49) respectively. Time–activity curves for breast tumour and background tissue are provided (Supplementary Fig. S1).

Seven first-order statistics were extracted (mean, standard deviation, min, max, range, skew, and kurtosis), and none showed a change in the variables, irrespective of whether they were in the early or late imaged breast tumour group (Fig. 2d).

### Voxel-wise analysis of [^18^F]ICMT-11 PET imaging data

As previously described [29], the analysis of apoptosis in PET imaging at the tumour level using a PET-based voxel intensity sorting (PVIS) approach allows for the overall spatial distribution of voxel intensities within the tumour to be assessed; higher ∆ intensity in treated tumours compared to baseline (right shift) is assigned PVIS apoptosis-dominant signature (PADS), while lower ∆ intensity (left shift) is assigned PVIS necrosis-dominant signature (PNDS).

**Fig. 3:**
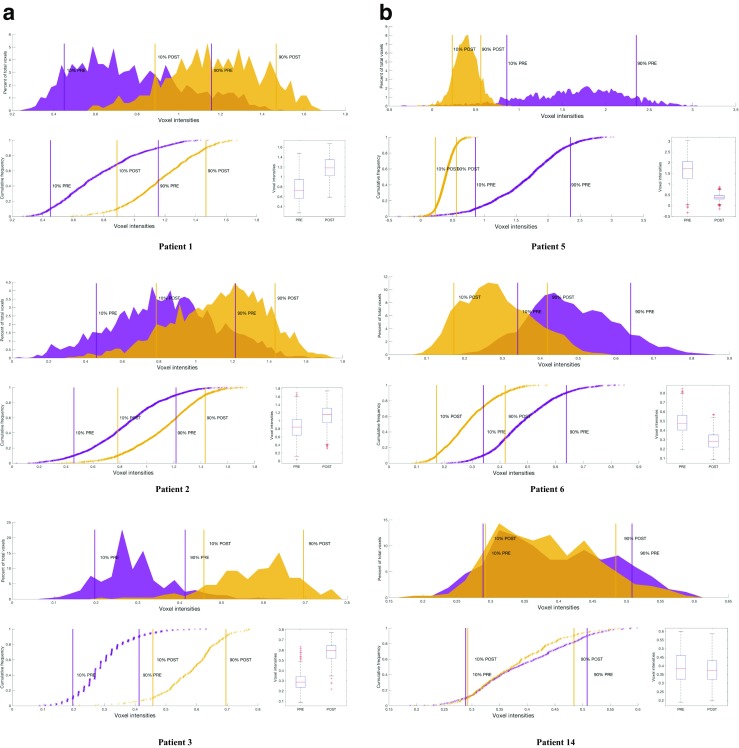

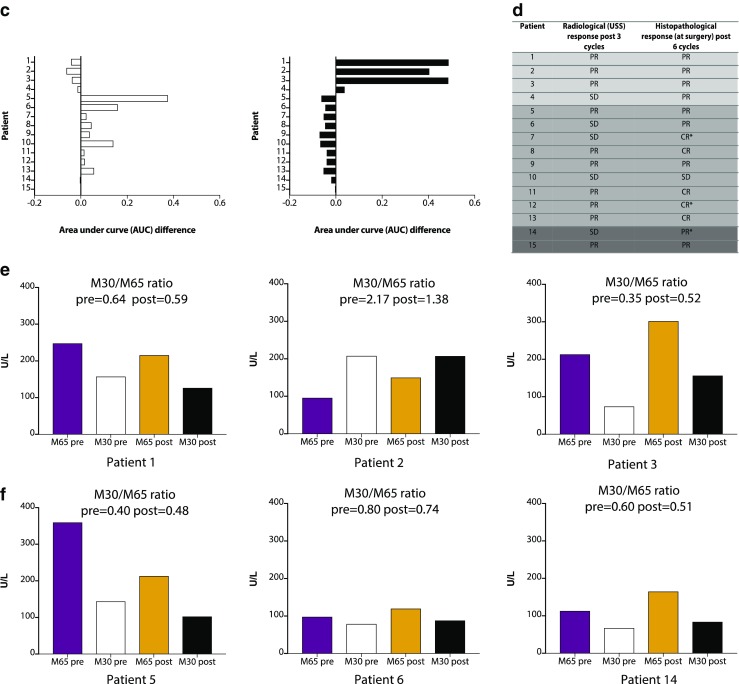
Voxel–wise tumour [^18^F]ICMT-11 intensity histogram analysis and blood cytokeratin-18 in all breast cancer patients. **a** PET-based voxel intensity sorting (PVIS) histogram analysis in patients 1, 2, and 3. All patients are late-imaged, (2–14 days) except patient 5* (early imaged 24–48 h). The intensities of all voxels within the tumour volume of interest (VOI) have been expressed as histogram plots of normalised voxel intensities pre-and post-chemotherapy. These patients demonstrated a predominant PVIS apoptotic signature with right shifts of all voxel intensities within the tumour VOI post-chemotherapy. **b** PVIS histogram analysis in patients 5 and 6, showing a predominant PVIS necrotic signature with shift in voxel intensities to the left. Patient 14, showing no dominant signature on PVIS histogram analysis. These results have been analysed statistically and are represented through box-plot diagrams (minimum, maximum, median, 10th and 90th percentile statistical parameters). The differences in AUC shifts noted in each patient are shown in **c**, demonstrating tumours with a predominant dominant PVIS apoptotic signature (*black*) or dominant PVIS necrotic signature (*white*), and **d** associated outcomes in all fifteen breast patients post 3 and 6 cycles of NCT. Patients are shown as having SD (stable disease), PR (partial response) or CR (complete response). * denotes patients who received trastuzumab alongside their 4th–6th cycle of chemotherapy. *Light grey shading* (patients 1–4, who showed a predominant PVIS apoptotic signature). *Mid-grey shading* (patients 5–13, who showed a predominant PVIS necrotic signature). *Dark grey shading* (patients 14 and 15, who showed neither a predominant apoptotic or necrotic shift). **e** Corresponding CK-18 analysis (M65 and M30) pre-and post-chemotherapy in patients 1, 2, and 3. Graphs highlight the variation in levels, with only patient 3 demonstrating an increase in post-chemotherapy levels of M65 and M30. **f** CK-18 (M65 and M30) analysis in patients 5 and 6, showing no clear increase post-chemotherapy, and patient 14, who despite showing no dominant signature on PVIS histogram analysis, was found to have an increase in M65 and M30 levels post-chemotherapy

Four patients (e.g., patients 1 and 2 in Fig. 2) displayed PADS (Fig. 3a); patient 3 in particular had a substantial right shift in voxel intensities. Mean percentage shift in voxel intensities for all four patients was 71%, and all had a PR after three cycles of chemotherapy on USS and after six cycles confirmed on histopathological correlation at surgery. The area under the curve (AUC) differences for all patients (numbered 1–15) are shown (Fig. 3c). Thus, it appears that despite a lack of change in SUV_60ave_ there were regional changes in tumour radiotracer uptake.

Of the 11 patients that did not demonstrate PADS, nine displayed ≥ 25% lower voxel intensities post-treatment indicating a PNDS. Examples of patients with PNDS — patients 5 (with post-treatment imaging at 24 h) and 6 (post-treatment imaging at 6 days) are shown in Fig. 3b. The mean percentage shift in voxel intensities was 68%; eight out of the nine patients had either a PR or CR post-chemotherapy, while one patient (patient 10) had SD (Fig. 3d), suggesting that either PADS or PNDS can be associated with response to therapy. The remaining two patients (exemplified by patient 14) showed no change in voxel intensity (Fig. 3b), despite showing PR to therapy. Notably, a snapshot of tumour [^18^F]ICMT-11 localisation is detected by our methodology; thus, presently we do not know whether the necrosis-dominant signature or indeed the no change in voxel intensities despite PR/CR, represents outright necrosis or a dynamic transition of cell death via an apoptosis dominant signature. In the cohort of patients studied, no patients demonstrated progressive disease.

### Cytokeratin-18 and caspase cleaved cytokeratin-18

All breast patients had blood taken for cytokeratin-18 (CK-18) analysis. Circulating full-length (M65) and caspase-cleaved CK-18 (M30) fragments were assessed by ELISA pre- and post-treatment at the time of the PET scan. Typical data are shown in Fig. 3e. Ratios of cleaved to total CK-18 varied considerably in this cohort of breast patients (Supplementary Fig. S2, Supplementary Table S2), with the lowest ratio post-chemotherapy 0.27 observed in patient 10, in whom there was no response to NCT. The median pre- and post-chemotherapy values of M65 were 141.5 U/l (range 50.3–359.7), and 180.8 U/l (range 66.9–369.7), and those for M30 were 78.8 U/l (range 32.2–207.9) U/L and 102.7 U/l (range 47.8–207.7) respectively. Overall M30 or M65 did not correlate with PADS or PNDS (Fig. 3f). Patient 3, who had the highest PADS, also showed the only consistent increase of M30/M65 ratio, increasing from 0.35 pre-chemotherapy to 0.52 post-chemotherapy, perhaps suggesting that the levels of apoptosis or necrosis were not sufficiently high to generate robust changes of M30/M65 in blood.

### Cleaved caspase-3 expression

**Fig. 4:**
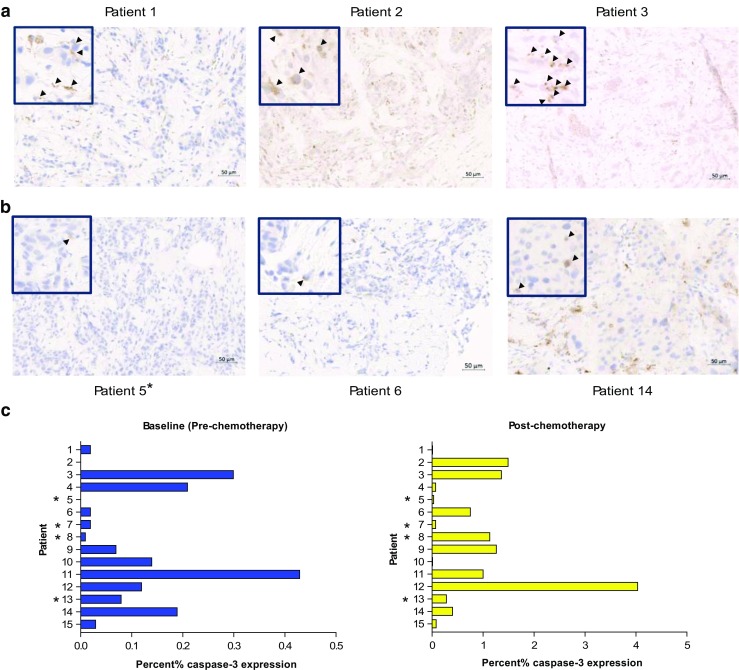
Cleaved caspase-3 expression in breast patients. **a** Expression of cleaved caspase-3 detected by immunohistochemistry in breast tissue taken by USS-guided biopsy post-chemotherapy in patients with a predominant PVIS apoptotic signature. **b** Similar cleaved caspase-3 expression in patients with a predominant PVIS necrotic signature no change on PVIS. *Arrows* (*black*) demonstrate cleaved caspase-3 staining on tissue biopsy post-chemotherapy. **c** Graph indicates the percentage (%) cleaved caspase-3 expression in breast tissue in all patients taken at baseline (*blue bars*) and post-chemotherapy (*yellow bars*). * denotes early imaged (24–48 h) breast patients

Acknowledging that pre- and post-treatment biopsies could have been taken from different parts of the tumour, we assessed if there was some general association between the PVIS data and histopathology in the breast patients. All diagnostic (pre-) and post-chemotherapy biopsies were stained for cleaved (active) capsase-3 expression. The diagnostic tumour blocks of two patients (patients 2 and 5) were not available. Typical cleaved caspase-3 immunostains in patients assigned PADS and PNDS are shown in Fig. 4a and b. Whole tissue mount analysis of percentage cleaved caspase-3 showed variable baseline and post-treatment expression. Cleaved caspase-3 expression was low at baseline with levels below 0.45%; expression increased from a mean of 0.13% (95% CI 0.12–0.13) to 0.81% (95% CI 0.79–0.83). Such low levels of caspase-3 activation despite a mean fold-change of 15.7% may prove difficult to detect by PET.

### Longitudinal studies in lung cancer patients

#### [^18^F]ICMT-11 uptake in lung tumours at baseline and post-chemotherapy

**Fig. 5:**
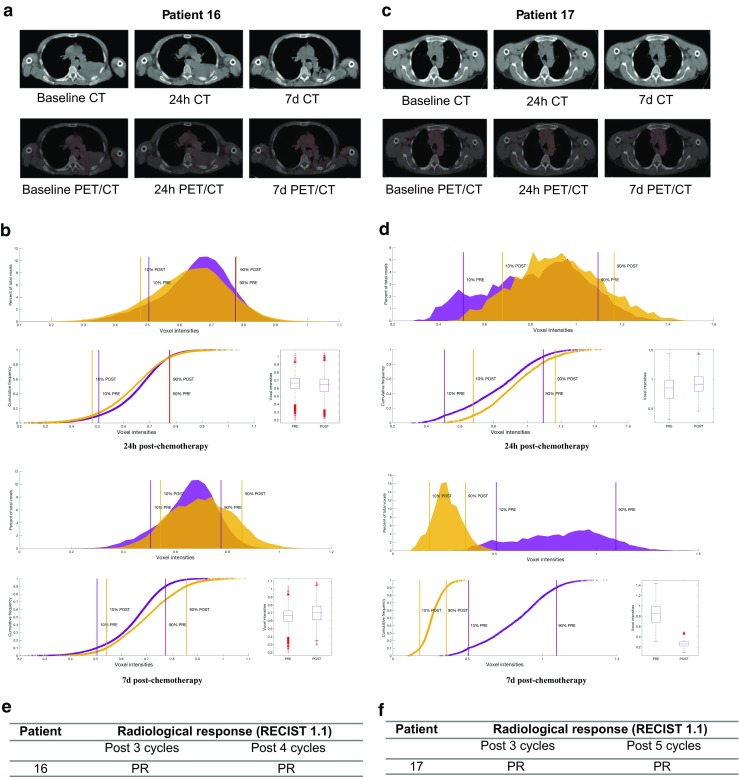
Detection of tumour cell death in lung cancer by [^18^F]ICMT-11 PET/CT. Patient 16 (**a**) and Patient 17 (**c**) axial CT and fused [^18^F]ICMT-11 PET/CT images of primary lung cancer at cancer at baseline, 24 h and 7 days post-chemotherapy. PVIS histogram analysis at 24 h and 7 days post-chemotherapy in patient 16 (**b**) and 17 (**d**), with clinical outcomes (**e** and **f**)

Three lesions from two lung cancer patients (a large left upper lobe tumour in patient 16; a primary lesion in the right upper thoracic mediastinum and a mediastinal lymph node in patient 17) were analysed at three time-points; the lesions were all visible on PET (Fig. 5a). The interval between baseline [^18^F]ICMT-11 PET/CT/MRI and start of chemotherapy in both lung cancer patients was between 2 and 8 days. Patients were scanned at 24 h and at 7 days after the first cycle of chemotherapy. Median (range) SUV_60ave_ and SUV_60max_ were 0.41 (0.33–0.61) and 0.85 (0.52–0.87) at baseline, 0.39 (0.35–0.62) and 0.87 (0.47–0.88) early after chemotherapy, and 0.45 (0.42–0.66) and 0.94 (0.64–0.98) late after chemotherapy. First-order statistics showed no differences in the features extracted. With regard to voxel-wise analysis, patient 16 demonstrated an initial PNDS shift followed by a PADS shift (61%) to higher voxel intensities at 7 days post-chemotherapy (Fig. 5a and b). Patient 17, had a PNDS in the primary thoracic lesion, at 24 h and 7 days post-chemotherapy (Fig. 5c and d). Both patients had a partial response to combination platinum-based chemotherapy (Fig. 5e and f).

#### Diffusion-weighted (DW) and dynamic contrast enhanced (DCE) MRI in lung cancer

(please see Supplementary results).

## Discussion

In the first patient study of the caspase 3/7-specific isatin sulphonamide PET radiotracer, we show that the lack of significant global tumour [^18^F]ICMT-11 dependent PET changes, despite some regional voxel changes, reflect a lack of significant apoptosis induction following chemotherapy. This study to our knowledge is the first to investigate the use of a caspase 3/7-specific PET radiotracer for imaging of chemotherapy-induced apoptosis in cancer.

Chemotherapy response has often been associated with cell death via apoptosis [21, 23, 24]; however, such studies have often been semi-quantitative and based on biopsy samples with variable output. Several studies have reported that spatial and temporal heterogeneity exist in breast and other cancers [31, 32]. Repeat biopsy of breast tissue at a single point in time is not representative of the whole tumour. Thus, approaches aiming to image whole-tumour apoptosis for the purposes of assessing therapeutic response (to chemotherapy, targeted therapies, and radiotherapy) were embraced. These imaging methods include MRI [20, 33], magnetic resonance spectroscopy (MRS) [34], USS [35], novel fluorescence imaging [36], scintigraphy [11, 18] and PET [6, 10, 16, 17]. Studies using [^99m^Tc]Annexin V, in particular, led the way for nuclear imaging of apoptosis [11, 19, 37].

In the current study, we report that caspase-3/7 activation as determined by [^18^F]ICMT-11 was not a dominant mechanism of pharmacological activity following chemotherapy in breast cancer. Two pieces of information enabled us to reach this conclusion. First, cytokeratin-18 analysis in blood samples, a method that has been previously reported to have high sensitivity and specificity for detecting apoptosis [38, 39], showed a positive change — increased M30/M65 ratio — only in one patient (patient 3). Indeed, Olofsson et al. indicated based on their M30/M65 data that FEC chemotherapy, also used by us, causes predominantly necrotic death compared for instance to taxane-based therapy [39]. Whether necrotic death was secondary to apoptosis was not determined. Furthermore, the absolute proportion of apoptotic cells in biopsy samples obtained from patients soon after [^18^F]ICMT-11 PET scanning was generally in the 1% range, albeit an increase from pre-therapy levels (Fig. 4c). This low proportion of apoptotic cells may not generally lead to detection by nuclear methods. While cytokeratin-18 methodology can be influenced by apoptosis of chemotherapy-sensitive healthy tissue [40], we were surprised by the low proportion of apoptosis from histology. In comparison to routine oncology nuclear medicine radiotracers, the (baseline) pre-chemotherapy uptake of [^18^F]ICMT-11 in tumour was low, with SUV_60max_ values around 1. With simultaneous acquisition of anatomical information, by CT in our case, segmentation of tumour is still possible and in view of this, low baseline uptake presumably representing the no/low apoptosis state is inconsequential. However, SUV parameters did not change following treatment, attributed to the low level of apoptosis seen in these tumours. In preclinical studies, [^18^F]ICMT-11 demonstrated high specificity to apoptosis versus necrosis [8, 29, 41]; thus, it is unlikely that the lack of changes is a reflection of lack of specificity. It is possible to rationalise a highly heterogeneous response of tumours to chemotherapy whereby clusters of tumours respond more avidly to the therapy than the bulk of the tumour. Indeed, we demonstrate this phenomenon in pre-clinical models of lung cancer imaged with [^18^F]ICMT-11 PET, whereby the global tumour tracer normalised uptake value returned no changes following effective therapy. In contrast, PVIS histogram analysis showed a clear right shift over 48 h post-treatment, with a 1.5-fold increase in the number of voxels having high intensity uptake [41]. Consequently, PVIS analysis allows extraction of the “cell-death” signal by allowing the capture of heterogeneous [^18^F]ICMT-11-detectable activated caspase-3/7 within the tumour, compared with simple uptake values derived from the volume of interest.

The over-reliance on voxel-based analysis for apoptosis data is predicated on pre-clinical studies for ICMT-11 [41] and clinical studies with [^18^F]ML-10 [16, 17], intimating a manner in which apoptosis data should be presented in view of its heterogeneous presentation. All patients, except one, responded to treatment; hence, we were unable to correlate PADS in particular, but also PANS to clinical outcome. All four patients with PADS responded to therapy; however, patients showing PANS or no change also responded to therapy, indicating that PADS is not a pre-requisite for response in this patient group. Notably, however, the only patient showing a positive M30/M65 also showed the highest PADS. Equally, we cannot infer a more appropriate time for [^18^F]ICMT-11 measurement, although the second window (2-14d) is perhaps more practical.

The lack of ‘non-responders’ is a limitation of our study; however, this could not be influenced due to the prospective nature of the study; thus, all outcomes were reported. The main aim — the investigation of the changes in ICMT-11 uptake and relationship with biochemical/histological caspase-3 activity — was, however, not compromised.

It is worth considering the difficulties within this study both from a logistics and scientific perspective. The timing of apoptosis has been elusive and fraught with difficulties when using functional imaging such as PET. Parton et al. [22] reported apoptosis in tissue biopsies rising within 24 h of chemotherapy in breast patients, a finding consistent with other studies [21, 23]. Due to logistics of imaging and availability of the patient, 24 h post-chemotherapy imaging was not feasible in all patients. In our study cohort, patients underwent imaging with [^18^F]ICMT-11 PET/CT at various time-points post-chemotherapy. Two patients imaged at 24 h post-chemotherapy failed to show an apoptotic dominant signature or significant increase in caspase-3 expression.

Apoptosis may not be the sole mechanism of cell death in treatment response. Although it is known to play a key role, cell death can occur by necrosis, mitotic catastrophe, senescence, autophagy, pyroptosis, and DNA damage [42]. As the majority of patients in our study showed a dominant necrotic or mixed apoptotic/necrotic signature phenotype, the balance between apoptosis and necrosis may be one in favour of the latter. This could in part, account for the lack of a predominant apoptotic signal on PET and histology.

Longitudinal, multi-parametric imaging studies in the lung cancer patients permitted us to verify simultaneously [^18^F]ICMT-11, ADC as a measure of cell death, and DCE-MRI as a measure of perfusion/permeability. The increase in ADC values seen with DW-MRI in patient 16, 24 h and 7 days post-chemotherapy, infers increased cell death-induced increases in water mobility as previously reported [43]. This is consistent with the increase in tumour [^18^F]ICMT-11 in the same patient. Conversely, the decrease in ADC variables in patient 17 may be linked to increases in ECM constituents [44] that can accompany response (necrosis, fibrosis, or mixed inflammatory infiltrate) and associated cell swelling [43]. Accordingly, in the two patients there appears to be congruence of [^18^F]ICMT-11 and ADC data. Significant changes in perfusion/permeability could perturb PVIS measurements. Assessment of the pharmacokinetics rate constant *K*^trans^ from the DCE-MRI study showed that perfusion/permeability dynamics could not explain the [^18^F]ICMT-11 dynamics within the time frame of the study. Beyond this proof of concept study, future prospective studies in a larger cohort should examine the role of [^18^F]ICMT-11 in assessing chemotherapy response, to also include a fair mix of responders and non-responders. The outcome of the lung cancer cohort study when confirmed in a larger cohort may support use of combined PET-MRI in monitoring ADC-detectable cell death and [^18^F]ICMT-11-detectable caspase-3/7 activation.

## Conclusion

In aggregate, initial studies using [^18^F]ICMT-11 were promising in preclinical and first-in-man healthy volunteer studies [8, 29, 41, 45]. We report the first use of [^18^F]ICMT-11 in a small cohort of patients diagnosed with breast or lung cancer and receiving first-line chemotherapy. The results show that only a small proportion of apoptosis was induced by drug treatment and that this level did not induce global changes in tumour [^18^F]ICMT-11 uptake. Voxel-wise analysis showed regional increases of [^18^F]ICMT-11 intensity regions in some tumours, and while patients having this phenotype responded to therapy, it was not an exclusive marker of response. Thus, tumour response could occur in the absence of predominant chemotherapy-induced caspase-3/7 activation measured non-invasively across entire tumour lesions in patients with breast and lung cancer.

## Electronic supplementary material

ESM 1 (DOCX 599 kb)
