# Supplementary material for: Clinical translation of [18F]ICMT-11 for measuring chemotherapy-induced caspase 3/7 activation in breast and lung cancer
Source: Eur J Nucl Med Mol Imaging. 2018 Sep 27;45(13):2285–99. doi: 10.1007/s00259-018-4098-9 (PMC6208806; doi:10.1007/s00259-018-4098-9)
Supplement: Supplementary file 1 — (DOCX 599 kb) [file 259_2018_4098_MOESM1_ESM.docx]

**SUPPLEMENTARY MATERIALS**

**Supplementary material and methods 1**

DW-MRI and DCE-MRI image analysis

**Supplementary material and methods 2**

Cytokeratin and blood radioactivity measurements

**Supplementary material and methods 3**

Cleaved (active) Caspase-3 immunohistochemistry

**Supplementary results**

**Supplementary figures**

Fig. S1 Time-activity curves (mean decay-corrected) of [^18^F]ICMT-11 for primary breast tumour and normal organs

Fig. S2 CK-18 analysis in all breast patients with M65, M30 and M30/M65 ratios

Fig. S3 Detection of tumour cell death in lung cancer by DW-MRI.

**Supplementary tables**

Table S1 DW-MRI scanning protocol

Table S2 CK-18 analysis with M65, M30 and M30/M65 ratio pre- and post-chemotherapy

Table S3 DW-MRI ADC Histogram analysis results of lung cancer patients 16 and 17

Table S4 DCE-MRI –Shutter speed model in lung cancer patients

**SUPPLEMENTARY MATERIALS**

**Supplementary material and methods 1**

**DW-MRI and DCE-MRI image analysis**

Lung MRI was performed on a moving-table 3T system (Siemens Verio with Syngo MR B17, Erlangen, Germany), using the body coil for transmission and a matrix phased array coil as receive coil. Axial slices were acquired during free-breathing for DW-MRI, while DW-MRI slice-matched *T*_2_-w imaging was also performed. Dynamic contrast enhanced MRI (DCE-MRI) was performed on the sagittal plane using a VIBE acquisition (0.1mmol/Kg) DOTAREM® (Supplementary Table S1).

ADC maps were generated in Matlab 15a (The MathWorks^(R)^) using monoexponential, non-linear fittings to the equation: *S*=*S*_0_·exp(-*b*·ADC) and all ten *b*-values (0, 10, 20, 30, 50, 80, 100, 150, 400 and 800 s/mm^2^). ADC values are expressed in μm^2^/s. For voxels in which the ADC calculation software failed to converge or returned negative values, the ADC value was set to zero.

All parametric maps were saved in DICOM format and imported into MIPAV image processing software (Medical Image Processing, Analysis and Visualisation, National Institutes of Health, US), where the regions of interest (ROIs) were drawn, with consideration to avoid regions affected by artefacts and partial volume effects. Multiple-slice ROIs were used (i.e. volumes of interest-VOIs), as opposed to single-slice ones for the analysis, to avoid observer bias and reduce ADC measurement variability [47]. Volumetric assessment is also expected to capture lesion heterogeneity more effectively than single-slice assessment and, as a result, is better suited for voxel-wise characterisation with histogram analysis. VOIs were drawn by an MRI physicist (IL) on the *b*=800 s/mm^2^ images and then copied to the ADC maps. Histogram analysis was then performed using in-house software developed in Matlab 15a (The MathWorks^(R)^), to calculate mean, skewness, kurtosis, 25^th^, 50^th^ and 75^th^ percentiles. Voxels with zero ADC values were excluded from the analysis.

For DCE-MRI, the shutter speed model was used. relaxation rate at baseline (*R_10_*) and relaxed signal (*M_0_*) were calculated, as 3dimensional maps, with the Ernst formula (assuming the echo time (TE)<< the transverse relaxation time (T2*)) using the set of spoiled gradient recalled echo (SPGRE) pre-contrast images acquired at different flip angles [48].

$$S(\alpha)=M_{0}\sin(\alpha)\frac{1-e^{-R_{10}\cdot T_{R}}}{1-\cos(\alpha)\cdot e^{-R_{10}\cdot T_{R}}}$$

where$\alpha_{i}$is the i^th^ flip angle and assumes values belonging to [2, 4, 8, 10, 14, 18, 22, 26] and$T_{R}$ is the repetition time set to 3.6 ms. The quantitative pharmacokinetic analysis of MRI contrast reagent bolus-tracking data were then modeled by the shutter speed pharmacokinetic model.

**The shutter speed model**

Shutter speed model accounts for finite transcytolemmal water exchange kinetics providing an additional pharmacokinetic parameter: the mean intracellular water molecule lifetime ($\tau_{i}$). It admits the system to leave the fast exchange limit (FXL), at rising values of concentration of the contrast media, and to enter the fast exchange regime (FXR), where the longitudinal water relaxation is characterized by a bi-exponential decay and the dependence between $R_{1}$ and concentration of contrast reagent, [CR], is no more linear:

$$\begin{matrix} R_{1}(t)=(1/2)\{2R_{1i}+r_{1o}[CR_{o}](t)+(R_{10}+R_{1i}+1/\tau_{i})/p_{o} \\ -[(2/\tau_{i}-r_{1o}[CR_{o}](t)-(R_{10}+R_{1i}+1/\tau_{i})/p_{o})^{2}+4(1-p_{o})/\tau_{i}^{2}p_{o}]^{1/2}\} \end{matrix}$$

$R_{1i}$ is the water rate constant in the absence of exchange, $r_{1o}$ is the interstitial CR relaxivity, $p_{o}$is the fraction of the extracellular tissue water and $\tau_{i}$ is the mean intracellular water molecule lifetime [s].

The time course of the contrast agent cannot be directly measured by the MRI experiment. Therefore, the Kety pharmacokinetic rate law is usually incorporated into the model in order to obtain the rate constants.

$$[CR_{o}](T)=K^{trans}\int_{0}^{T} [CR_{p}](t)e^{-\frac{K^{trans}}{v_{e}}(T-t)}dt$$

$K^{trans}$is the first order rate constant for plasma to interstitium CR transport ([1/min]) and $v_{e}$is a measure of the EES volume fraction. The ratio between $K^{trans}$ and $v_{e}$ results in the third pharmacokinetic parameter$K_{ep}$, that is the back flux rate constant [1/min]. $[CR_{o}]$ and $[CR_{p}]$ are related to the concentration of CR in the 'outside' space $[CR_{o}]$ and in the plasma $[CR_{p}]$, also called the arterial input function (AIF).

VOIs were drawn by a single investigator (SD) on the dynamic scan of the lung patients DCE MRI data using the Analyze software (Biomedical Imaging Resource, Mayo Foundation, Rochester, MN). All 3D parametric maps were generated in Matlab R2015a (The MathWorks^(R)^) using a standard non-linear least square curve fitting algorithm. Mean, standard deviation, kurtosis and skewness values were evaluated for each parameter. Voxels with biologically implausible results were excluded from the analysis (e.g. $K^{trans}$ > 5 1/min, $v_{e}$ > 1, $\tau_{i}$ > 3 s as any negative value). Implicit in the analysis is the requirement of the AIF which, in this study, has been evaluated, for each subject, drawing a region of interest in the aorta.

**Table S1**

**Lung MRI imaging protocol**

|  | *T*_2_-w MRI | DW-MRI | DCE-MRI |
| --- | --- | --- | --- |
| Sequence type | HASTE ^c^ (axial) | SS SE EPI ^a^ (axial) | VIBE (sagittal) |
| FOV (mm) | 380×267 | 380×285 | 350×306 |
| Matrix size | 256×180 | 128×128 | 128×128 |
| No of slices/ thickness/spacing (mm/%) | 30/5/20% | 10/5/0% | 32/5/20% |
| *TR* (ms) | 1400 | 1100 | 3.6 |
| *TE* (ms) | 55 | 96 | 1 |
| Bandwidth (Hz/pixel) | 781 | 1502 | 800 |
| Flip Angle (°) | 160 | 90 | 14 |
| *N*_A_ | 1 | 1 | 1 |
| Fat suppression | None | SPAIR | Q-fat. Sat. |
| *b*-values (s/mm^2^) | N/A | 0,10,20,30,50,80,  100,150,400,800 | N/A |
| Parallel Acquisition | GRAPPA 2 | GRAPPA 2 | GRAPPA 2 |
| Half Fourier | 5/8 | - | 6/8 |
| # measurements | N/A | N/A | 100 |
| *T*_A_ (min) | 0:42 | 0:29 | 6:41 |

^a^ SS SE EPI=single-shot spin echo planar imaging, ^b^ VIBE=3D volumetric interpolated breath-hold examination,

^c^ HASTE=half-Fourier acquisition single-shot turbo spin-echo, ^d^ STIR=short inversion time inversion recovery,

^e^ GRAPPA=generalised autocalibrating partially parallel acquisition.

**Supplementary material and methods 2**

**Cytokeratin-18 measurements**

The M65 and M30 assays were performed under dedicated Good Clinical Laboratory Practice conditions [49]. Two samples were taken from each patient to cross-check for inherent variability. To ensure reproducibility inter-assay and intra-assay sampling was performed. Background variation cut-off for M65 and M30 antigens is taken as ±10% as per manufacture guidelines [40].

**Supplementary material and methods 3**

**Cleaved (active) Caspase-3 immunohistochemistry**

Briefly, tissue was fixed in formalin and embedded in paraffin, sectioned (5μm slices) and stained as per manufacturer instructions, for cleaved (active) caspase-3 using Cleaved Caspase-3 (Asp 175) monoclonal antibody (Cell Signaling Technology, 1: 200 dilution).

Whole mounts were scanned using the Axio Scan.Z1 (Zeiss) and images were viewed using Axio Scan.Z1’s ZEN imaging software. Tissue samples were initially scored manually by an independent histopathologist who was blinded to all clinical data, using the immunohistochemical score (IHS), as described previously [50]. Scoring was also performed using Image J software and pre-defined algorithms for distinguishing apoptosis from necrosis.


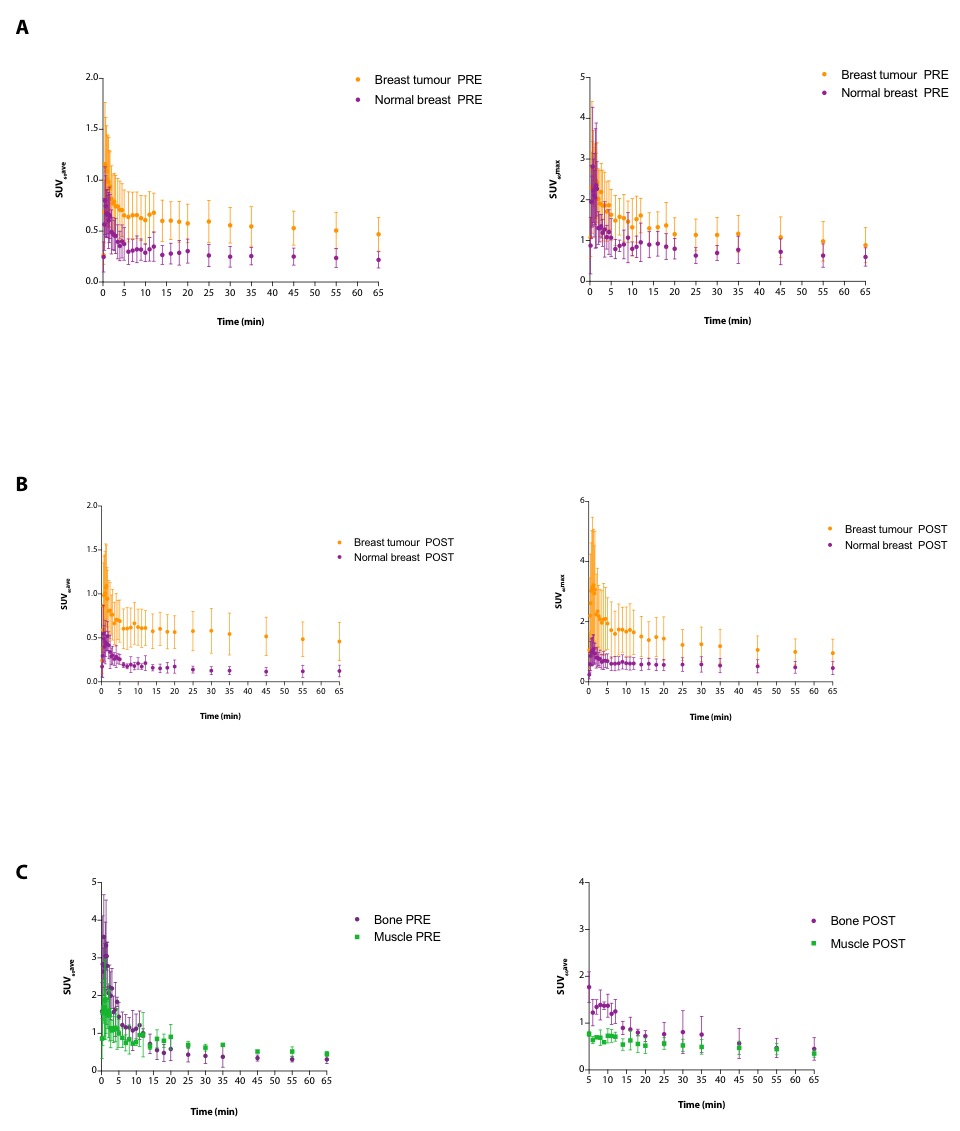


**Fig. S1. Time-activity curves (mean decay-corrected) of [^18^F]ICMT-11 for primary breast tumour and normal organs.**

Mean SUV_60ave_ and SUV_60max_ are shown over 65 min of dynamic imaging of breast tumours (**A**) pre- and (**B**) post chemotherapy. (**C**) Normal organs are shown pre- and post-chemotherapy.

**Fig. S2. Box-plot graphs showing CK-18 fragment analysis (M65 and M30) pre- and post-chemotherapy values.** Box-plots demonstrate the minimum, maximum range, and median values for all breast patients. The lower limit of quantification for M65 and M30 interassay is 11 U/L and 40 U/L respectively.

**Table S2.**

**Patient CK-18 analysis with M65, M30 and M30/M65 ratio pre- and post-chemotherapy**

|  | | M65 | | | M30 | | | M30 /M65 Ratio | |
| --- | --- | --- | --- | --- | --- | --- | --- | --- | --- |
| Patient | **Pre** | | **Post** | **Pre** | | **Post** | **Pre** | | **Post** |
| 1 | 247.79 | | 215.24 | 157.40 | | 126.55 | 0.64 | | 0.59 |
| 2 | 95.88 | | 150.09 | 207.92 | | 207.65 | 2.17 | | 1.38 |
| 3 | 213.40 | | 302.12 | 74.12 | | 156.60 | 0.35 | | 0.52 |
| 4 | 279.57 | | 224.54 | 164.31 | | 136.98 | 0.59 | | 0.61 |
| 5 | 359.66 | | 213.33 | 144.05 | | 102.72 | 0.40 | | 0.48 |
| 6 | 98.02 | | 119.73 | 78.84 | | 88.31 | 0.80 | | 0.74 |
| 7 | 190.82 | | 294.68 | 116.78 | | 149.05 | 0.61 | | 0.51 |
| 8 | 141.48 | | 369.06 | 124.90 | | 206.24 | 0.88 | | 0.56 |
| 9 | 121.74 | | 140.36 | 98.43 | | 104.71 | 0.81 | | 0.75 |
| 10 | 304.61 | | 211.83 | 66.26 | | 57.25 | 0.22 | | 0.27 |
| 11 | 50.33 | | 66.91 | 32.15 | | 47.77 | 0.64 | | 0.71 |
| 12 | 84.44 | | 84.44 | 47.21 | | 50.12 | 0.56 | | 0.59 |
| 13 | 169.94 | | 180.82 | 74.07 | | 64.07 | 0.44 | | 0.35 |
| 14 | 113.15 | | 164.93 | 67.46 | | 84.10 | 0.60 | | 0.51 |
| 15 | 72.23 | | 139.22 | 74.44 | | 74.92 | 1.03 | | 0.54 |

Light grey shading (patients 1-4, found to show a predominant apoptotic signature).

Mid-grey shading (patients 5-13, found to show a predominant necrotic and/or necrotic/apoptotic signature).

Dark grey shading (patients 14 and 15, found to show neither a predominant apoptotic nor necrotic shift).

**Supplementary results**

**Diffusion-weighted (DW) and dynamic contrast enhanced (DCE) MRI in lung cancer.**

The diffusion of water molecules embodied in the variable Apparent Diffusion Coefficient (ADC; mean and percentiles), increased in patient 16 but not in patient 17 (Supplementary Table S3) consistent with cell death-related increase in extracellular space in the former after the initiation of therapy [31] (Fig.S3A and B). Histogram analysis demonstrated increase in skewness and kurtosis in patient 16, particularly at 7d, while these variables decreased or were unchanged in patient 17 (Fig.S3C and D). A reduction of ADC and skewness could be related to increases in extracellular matrix (ECM) constituents [32], which introduces additional obstructions and hydrogen-bonding sites to the tumour microenvironment, thus reducing the ADC.

DCE-MRI pharmacokinetic analysis using the shutter speed model was conducted to verify whether large changes in perfusion/permeability accompanied [^18^F]ICMT-11. *K*^trans^ is a variable that reflects permeability and blood flow, and measured as the accumulation of contrast agent into the extravascular-extracellular space. In general, *K*^trans^ increased at 24h followed by a decrease to below baseline values at 7d (Supplementary Table S4), suggesting that changes in [^18^F]ICMT-11 tumour uptake in the lung patients could not be explained by perfusion/permeability changes. Other DCE variables including *K*_ep_, V_e_ and $\tau_{i}$ (mean and histogram values) are summarised in Supplementary Table S4.

**Table S3**

**ADC histogram analysis results of lung cancer patients 16 and 17**

|  | Patient 16 | | | | | | |
| --- | --- | --- | --- | --- | --- | --- | --- |
|  | **#Voxels** | **Mean** | **Skew** | **Kurtosis** | **25^th^ perc.** | **50^th^ perc.** | **75^th^ perc.** |
| Baseline | 10274 | 998.2 | 0.27 | 4.71 | 881.0 | 995.0 | 1111.0 |
| 24 h | 10261 | 1109.0 | 0.67 | 5.74 | 986.0 | 1099.0 | 1214.0 |
| 7 d | 3781 | 1343.2 | 2.14 | 26.5 | 1105.0 | 1309.0 | 1546.0 |
|  | **Patient 17** | | | | | | |
|  | **#Voxels** | **Mean** | **Skew** | **Kurtosis** | **25^th^ perc.** | **50^th^ perc.** | **75^th^ perc.** |
| Baseline | 2157 | 1743.5 | | 0.68 | | --- | | 2.79 | 1263.7 | 1614.0 | 2131.5 |
| 24 h | 1976 | 1315.5 | 0.25 | 3.12 | 1104.0 | 1291.5 | 1521 |
| 7 d | 1781 | 1459.5 | 0.15 | 3.54 | 1264.7 | 1454 | 1645 |

**
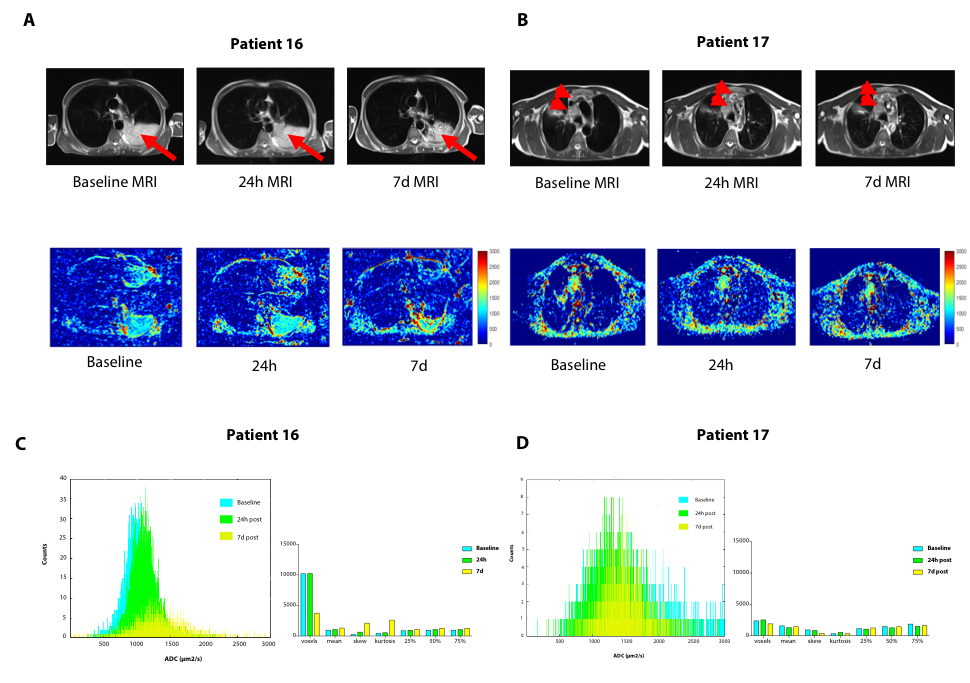
**

**Fig. S3. Detection of tumour cell death in lung cancer by DW-MRI**.

(**A and B**) DW-MRI images for patient 16 and 17 at baseline, 24h and 7d post-chemotherapy. Corresponding apparent diffusion coefficient (ADC) maps in jet colour scale (µm^2^/s) are shown. (**C** and **D**) ADC tumour histograms in patient 16 and 17 depicting the distribution of the voxels as per their intensities at baseline, 24h and 7d post-chemotherapy with histogram analysis using in-house software developed in Matlab 15a (The MathWorks^(R)^), to calculate mean, skewness, kurtosis, 25^th^, 50^th^ and 75^th^ percentiles. Voxels with zero ADC values were excluded from the analysis.

**Table S4**

**DCE-MRI histogram analysis values of lung cancer patients**

| SHUTTER SPEED MODEL | | | | | | | | | | | | | | | | | |
| --- | --- | --- | --- | --- | --- | --- | --- | --- | --- | --- | --- | --- | --- | --- | --- | --- | --- |
| Patient 16 | | | | | | | | | | | | | | | | | |
|  | $K^{trans}$ | | | | $K_{ep}$ | | | | $v_{e}$ | | | | $\tau_{i}$ | | | | |
|  | Mean | Std | Kurtosis | Skewness | Mean | Std | Kurtosis | Skewness | Mean | Std | Kurtosis | Skewness | Mean | | Std | Kurtosis | Skewness |
| Baseline | 0.7618 | 1.119 | 5.782 | 1.9051 | 1.5289 | 1.3801 | 2.4933 | 0.8526 | 0.7898 | 0.1412 | 10.9927 | -2.143 | 0.2259 | | 0.3244 | 18.0465 | 3.4828 |
| 24 h | 0.943 | 1.2665 | 4.4434 | 1.6544 | 2.0553 | 1.5184 | 1.7585 | 0.4839 | 0.8101 | 0.1088 | 8.7422 | -1.1508 | 0.1814 | | 0.2104 | 28.6669 | 4.0826 |
| 7 d | 0.5755 | 0.7013 | 12.0182 | 2.8014 | 0.7832 | 0.8665 | 7.7588 | 2.192 | 0.6773 | 0.205 | 2.091 | -0.3006 | 0.1974 | | 0.1318 | 4.5997 | 0.4533 |
| Variation | -0.3897 | -0.4462 | 1.7047 | 0.6933 | -0.6189 | -0.4293 | 3.4121 | 3.5298 | -0.1639 | 0.8841 | -0.7608 | -0.7387 | 0.0882 | | -0.3735 | -0.8395 | -0.8889 |
| Patient 17 | | | | | | | | | | | | | | | | | |
|  | $K^{trans}$ | | | | $K_{ep}$ | | | | $v_{e}$ | | | | | $\tau_{i}$ | | | |
|  | Mean | Std | Kurtosis | Skewness | Mean | Std | Kurtosis | Skewness | Mean | Std | Kurtosis | Skewness | Mean | | Std | Kurtosis | Skewness |
| Baseline | 1.2622 | 1.1958 | 4.0168 | 1.3075 | 1.9814 | 1.2979 | 1.9003 | 0.2926 | 0.7444 | 0.2378 | 2.8289 | -1.088 | 0.0871 | | 0.3547 | 176.375 | 12.6254 |
| 24 h | 1.1808 | 1.1698 | 3.7583 | 1.3269 | 1.4549 | 0.9646 | 4.1663 | 1.0752 | 0.6971 | 0.2522 | 2.1194 | -0.7984 | 0.1033 | | 0.0749 | 4.8202 | 0.9029 |
| 7 d | 1.1162 | 1.3229 | 3.7701 | 1.4103 | 0.8086 | 0.7431 | 4.8047 | 1.6506 | 0.681 | 0.2859 | 3.0041 | -1.0872 | 0.1566 | | 0.2904 | 11.0662 | 2.8419 |
| Variation | -0.0547 | 0.1308 | 0.0031 | 0.0628 | -0.4442 | -0.2296 | 0.1532 | 0.5351 | -0.0231 | 0.1336 | 0.4174 | 0.3617 | 0.5159 | | 2.8771 | 1.2957 | 2.1475 |
